# Striatal Injury Induces Overall Brain Alteration at the Pallial, Thalamic, and Cerebellar Levels

**DOI:** 10.3390/biology11030425

**Published:** 2022-03-10

**Authors:** Kristina Lukacova, Julie Hamaide, Ladislav Baciak, Annemie Van der Linden, Lubica Kubikova

**Affiliations:** 1 Institute of Animal Biochemistry and Genetics, Centre of Biosciences, Slovak Academy of Sciences, 840 05 Bratislava, Slovakia; 2 Bio-Imaging Laboratory, Faculty of Pharmaceutical, Biomedical and Veterinary Sciences, University of Antwerp, B-2610 Antwerp, Belgium; julie_hamaide@uantwerpen.be (J.H.); annemie.vanderlinden@uantwerpen.be (A.V.d.L.); 3 Central Laboratories, Faculty of Chemical and Food Technology, Slovak University of Technology, 812 37 Bratislava, Slovakia; ladislav.baciak@stuba.sk

**Keywords:** songbird, Area X, neural plasticity, deep cerebellar nuclei, FoxP2, PNNs

## Abstract

**Simple Summary:**

Magnetic resonance imaging showed that striatal injury leads to structural changes within several brain areas. Here, we specify these changes via gene expression of synaptic plasticity markers, neuronal markers, assessing the number of newborn cells as well as cell densities. We found that the injury resulted in long-lasting modifications involving plasticity and neural protection mechanisms in areas directly as well as indirectly connected with the damaged striatum, including the cerebellum.

**Abstract:**

The striatal region Area X plays an important role during song learning, sequencing, and variability in songbirds. A previous study revealed that neurotoxic damage within Area X results in micro and macrostructural changes across the entire brain, including the downstream dorsal thalamus and both the upstream pallial nucleus HVC (proper name) and the deep cerebellar nuclei (DCN). Here, we specify these changes on cellular and gene expression levels. We found decreased cell density in the thalamic and cerebellar areas and HVC, but it was not related to neuronal loss. On the contrary, perineuronal nets (PNNs) in HVC increased for up to 2 months post-lesion, suggesting their protecting role. The synaptic plasticity marker Forkhead box protein P2 (FoxP2) showed a bi-phasic increase at 8 days and 3 months post-lesion, indicating a massive synaptic rebuilding. The later increase in HVC was associated with the increased number of new neurons. These data suggest that the damage in the striatal vocal nucleus induces cellular and gene expression alterations in both the efferent and afferent destinations. These changes may be long-lasting and involve plasticity and neural protection mechanisms in the areas directly connected to the injury site and also to distant areas, such as the cerebellum.

## 1. Introduction

**Figure 1:**
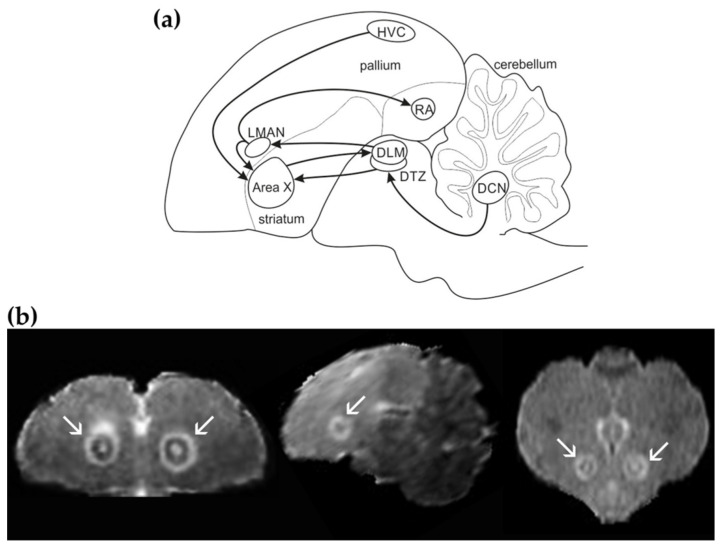
Brain circuitry and Area X lesion. (**a**) Schematic sagittal drawing of the song control system (SCS) in the songbird brain with delineated brain areas relevant to this study. Arrows are oriented in the direction of neuronal projections. Area X—vocal region of the striatum, DCN—deep cerebellar nuclei, DLM—the dorsolateral nucleus of the anterior thalamus, DTZ—dorsal thalamic zone, HVC—vocal nucleus (abbreviation used as proper name), LMAN—the lateral magnocellular nucleus of the anterior nidopallium, RA—robust nucleus of the arcopallium. The connectivity is based on previous studies [10,19]. (**b**) Magnetic resonance T2-weighted image of Area X on day 10 after excitotoxic injury showed in 3-orthogonal views (coronal, sagittal, and horizontal from left to right). The lesion spreads over the entire Area X. The hyper-intense border line due to edema is indicated by white arrows.

The song control system (SCS) in songbird forms pathways necessary for learning, production, and perception of learned vocalization [1,2,3,4,5,6,7]. The main vocal pathways consist of the anterior forebrain pathway (AFP) that enables song learning and maintenance, and the song motor pathway (SMP), important for the act of singing [2,5,6,7,8,9,10,11]. The AFP in the songbirds includes the pallio-basal ganglia-thalamo-pallial loop and connects the pallial nucleus HVC (proper name) to the striatal nucleus Area X to the dorsolateral nucleus of medial thalamus (DLM) to the robust nucleus of the arcopallium (RA) (Figure 1a). This loop has been compared to the mammalian premotor cortico-basal ganglia-thalamo-cortical loops [12,13]. Other parts of the SCS also have their homological parts in humans [8,14,15].

The involvement of the cerebellum in language was first noticed in humans with speech deficits in 1917 ([16] reviewed in [17,18]), but it has not been traditionally associated with learned song production in songbirds. The anatomical connection of the cerebellum with SCS has been emphasized only recently. Since damage in deep cerebellar nuclei (DCN) impairs juvenile vocal learning, the cerebellar role in learned vocal communication seems to be in sensorimotor learning [10,19]. Its role in the processing of sensorimotor information, however, remains elusive [19]. DCN sends projections to the dorsal thalamic zone (DTZ) around DLM and DTZ, and in turn, innervates Area X (Figure 1a). Electrical stimulation in DCN drives responses in the pallidal projection neurons in Area X [19]. Neurotoxic injury in the striatal part of the pallio-basal ganglia-thalamo-pallial loop, Area X, leads to micro and macrostructural changes in DCN as well as in areas directly connected to the damaged area, vocal nucleus HVC, and DLM, for up to 4 months [20]. Further, Area X injury results in the increased syllable repetition and song tempo in adult zebra finches (*Taeniopygia guttata*) [9] and Bengalese finches (*Lonchura striata domestica*) [21] and is decreased within syllable variation in fundamental frequency [22]. Area X regenerates mostly over the period of 1–2 months [9,23]. The brain alterations associated with the loop disruption last for several months [20]. The changes following Area X damage were reported previously. In this study, we focused on the identification of these alterations.

The majority of neurons in Area X constitute medium spiny neurons (MSN) [24]. This neuronal type is recruited during adult neurogenesis [25,26,27,28] as well as during the regeneration following the Area X damage [9]. MSN expresses dopamine and cAMP-regulated phosphoprotein of 32 kDa (DARPP-32) [26] as well as the transcription factor Forkhead box protein P2 (FoxP2) [29]. FoxP2 can have a developmental role, as its expression level is associated with neuronal differentiation and spine density [30,31,32] and promotes sharpening synaptic plasticity [33,34,35]. Developmental and seasonal changes in Area X suggest that elevated levels of FoxP2 occur during vocal learning and unstable song [36]. MSN maturation, activation during singing, and social context-dependent undirected singing are associated with a relatively decreased expression of FoxP2 [37,38]. FoxP2 mutation is associated with impairment in vocalization, speech, and other language disorders [35,39,40]. These findings suggest the role of FoxP2 in synaptic plasticity.

Other elements controlling brain plasticity are perineuronal nets (PNNs). A high level of their expression was observed before the peak of postnatal synaptogenesis, which suggests the importance of PNNs for the formation of synapses in immature, developing brains [41]. PNNs components play a key role as a physical barrier to control changes in the formation of a new connection between neurons also in the mature brain [42]. One of the important roles of PNNs is to stabilize the extracellular neuronal matrix and protect neurons from the influence of harmful agents. They preferentially surround inhibitory GABAergic neurons, which normally initiate a sensitive period [43], and parvalbumin (PV+) interneurons [44]. The function of PNNs is associated mainly with onset as well as the offset of the (developmental) critical period of neuroplasticity [45,46,47] and stabilizing synaptic connectivity [44,45,48]. In songbirds, PNNs density increases with the developmental stage [45,49], and their expression at the level of the SCS is different in males and females [46,50,51]. Degradation of PNNs can reopen critical windows of experience-dependent plasticity in the visual cortex in rats [52]. Enzymatic degradation of PNNs in songbirds, however, was not necessary to maintain the crystalized song [53].

Here we investigated and identified some of the changes that occur on the protein expression level after the impairment of the striatal part of the pallio-basal ganglia-thalamo-pallial loop between 1 month and 4 months. We focused on the expression of FoxP2, PNNs, and PV, but we also examined the incorporation of newborn cells.

## 2. Materials and Methods

### 2.1. Animals

Thirty-three adult male zebra finches (*Taeniopygia guttata*) from our colony in the Centre of Biosciences, Slovak Academy of Sciences, Institute of Animal Biochemistry and Genetics in Bratislava, were used in this study. All experimental procedures were approved by the State Veterinary and Food Administration of the Slovak Republic (permit number 2982/17-221/3) and by the Committee of Animal Care and Use at the University of Antwerp, Belgium (permit number: 2015-03). Birds were housed in the colony under 14:10 L:D cycle, temperature 27 ± 3 °C, humidity 55 ± 4%, with food, water, grit, and cuttlebone available ad libitum.

### 2.2. Striatal Lesion

Bilateral neurotoxic lesions of the striatal vocal nucleus Area X were created by stereotaxic surgery under an isoflurane inhalation anesthesia (concentration 1.5–2.7%; flow 0.8–1 L/min; Vetpharma Animal Health, Spain). We used a local anesthetic (Mesocain, Zentiva, SK) before the skin cut and defined zero point as a point where the two hemispheres and the cerebellum meet. In each hemisphere, we followed coordinates: anterior-posterior 4.5–5.0 mm, medial-lateral −1.3/+1.3 mm, and dorsal-ventral −3.5 mm with the beak at a 20° angle to avoid disrupting the lateral magnocellular nucleus of the anterior nidopallium (LMAN; vocal nucleus). Lesions were created with ibotenic acid (diluted to 1% in sterile saline, pH = 7.4–7.5; Tocris, UK) using the Nanoject II pressure injector (Drummond Scientific, Broomall, PA, USA) to inject 46nl into Area X three times (total 138 nl) bilaterally. We waited 2–5 min between the injections to prevent leakage of the neurotoxin. Birds recovered under an infrared heating lamp and were transferred to the home cages the next day. The birds were administered with a marker of the newborn cells 5-bromo-2′-deoxyuridine (BrdU; Sigma-Aldrich, St. Louis, MO, USA; concentration 10 mg/mL; dose 50 mg/kg i.m.) during seven consecutive days after the surgery, consistently at the same time before noon.

### 2.3. Experimental Timeline & Brain Scanning

The experimental animals were divided into 6 groups based on the time of sacrifice after the Area X lesion—8 days (8D, n = 5), 1 month (1M, n = 5), 2 months (2M, n = 7), 3 months (3M, n = 4), and 4 months (4M, n = 7). Seven birds 4M were used from our previous study [20]. The control group (baseline, n = 5), consisted of sham-operated birds that underwent the same surgical procedure as the other experimental groups but without the neurotoxin injection. The baseline group was sacrificed on the eighth day following the sham operation and one day after the last BrdU administration. All lesioned birds underwent magnetic resonance imaging (MRI) 8–10 days after Area X damage to confirm the proper lesion position. Scanning details of the birds from the 4M group are described in our previous study [20]. All the other groups were scanned using a 4.7 Tesla MRI scanner (Agilent, Yarton, UK) equipped with a 400 mT/m gradient insert. A quadrature volume coil transmitter with an internal diameter of 72 mm (Rapid Biomed, Rimpar, Germany) and a two-channel anatomically shaped surface coil receiver (STARK Contrast, Erlangen, Germany) were used for signal detection. T2-weighted 3D images were acquired by a 3D fast spin-echo sequence with the following parameters: TR/TEeff/NEX = 2000 ms/80 ms/1, echo train length 8, echo spacing 10 ms, FOV 30 × 30 × 20 mm^3^, matrix size 256 × 256 × 128, giving an isometric resolution of 156 μm. The scanning time was 1 h 16 min. During the MR scanning, the birds were under isoflurane anesthesia. The initial dose was 2.5% and the maintenance dose was 1.5–1.7%, keeping the respiration rate above 50 bpm. The temperature in the scanner was maintained at 39 °C by warm air and the respiration rate was constantly monitored (SA instruments, Stonybrook, NY, USA). The birds recovered under an infrared heating lamp and 4 h later they were transferred to the home cages.

### 2.4. Behavioral Analysis

The home cages were in sound-attenuating boxes where the songs of the males were recorded. The cages were divided into two compartments. Every male was placed into one compartment and a female was placed in the second compartment once a week to enrich the social context. The other conditions, such as food, water, light, temperature, and humidity, were the same as in the colony. On the last day, when the lights turned on, the birds could sing for one hour after the onset of the first song. Then the birds received a lethal dose of anesthetic followed by the transcardial perfusion as described below. The song recordings were obtained and processed by the Sound Analysis Pro software [54]. The number of song motifs sung 1 h before the perfusion was counted. The song motif is defined as the sequence of syllables, and this sequence can be repeated several times in the song.

### 2.5. Tissue Processing & Immunohistochemical Staining

The transcardial perfusion started with a 2 min infusion of 0.1 M phosphate buffer saline (PBS) and was followed by a 20 min infusion of 4% paraformaldehyde (PFA) in 0.1 M PBS. The brains were dissected and post fixed for 5 h in 4% PFA, immersed for 12 h in 20% sucrose and 24 h in 30% sucrose, frozen in OCT Compound (Sakura, Japan), and stored at −20 °C until the brains were processed. Sagittal sections 20 µm thick were cut using a cryocut (Leica, Microsystems, Wien, Austria) at −20 °C, mounted immediately on silanated slides, and stored at −20 °C.

Cells were visualized by the immunohistochemical (IHC) staining method. We stained for neurons (NeuN), PNNs, PV, cell division (BrdU), FoxP2, and young neurons (doublecortin, DCX). The brain sections were fixed on slides for 3 min in 4% PFA and washed 3 times for 2 min in 0.1 M PBS at room temperature. For BrdU visualization, the sections were incubated in 2 M HCl at 37 °C for 7 min to denature the DNA. The reaction was stopped by incubation in 0.1 M borate buffer (pH 8.0) at room temperature for 4 min. The sections were then washed 3 times in PBS. For all stainings, non-specific binding was reduced by incubation in blocking solution containing 1% bovine serum albumin (BSA, Sigma, Saint-Louis, MO, USA) and 0.2% Triton X-100 in 0.1 M PBS for 1 h. Afterward, the sections were incubated at 4 °C for 48 h with primary antibodies diluted in the blocking solution. The primary antibodies were the mouse anti-NeuN diluted 1:200 (Millipore, cat. # MAB377), mouse anti-PNNs diluted 1:150 (anti-chondroitin sulfate, Sigma, cat. # C8035), rabbit anti-PV diluted 1:200 (Abcam, cat. # ab11427), rat polyclonal anti-BrdU diluted 1:250 (Accurate Chemicals, cat. # OBT 0030), goat anti-FoxP2 diluted 1:1000 (Abcam, cat. # ab1307), rabbit anti-DCX diluted 1:500 (Sigma, cat. # AV41333). Then, the sections were washed 3 times in PBS at room temperature and incubated with the secondary antibodies for 2 h in the dark. The sections were labeled by donkey anti-mouse IgG conjugated with Alexa 488 (for NeuN, PNNs; all secondary antibodies conjugated with Alexa fluorophore were from Molecular Probes, Waltham, MA, USA), goat anti-rabbit IgG conjugated with Alexa 594 (for PV, DCX), donkey anti-goat IgG conjugated with Alexa 488 (for FoxP2), and donkey anti-rat Alexa 568 (for BrdU). All secondary antibodies were diluted 1:250 in the blocking solution. Then, the sections were washed 3 times in PBS and rinsed in deionized water. They were coverslipped with the Vectashield mounting medium with or without 4,6-diamidin-2-phenylindol (DAPI; Vector Laboratories, Burlingame, CA, USA).

### 2.6. Image & Statistical Analysis

The IHC and MRI scans were post-processed using the free software package Fiji [55]. Before the image analysis of MR data, the data were zero-filled to 256 × 256 matrix sizes, giving a spatial resolution of 78 mm. The down-sampling was used to increase the contrast to noise ratio in the Z direction. The lesion volume was calculated by the same method as we published previously [23].

The IHC staining was evaluated from photomicrographs taken with the Leica DM 5500 microscope, Leica DFC 340 FX camera, and Leica Microsystems LAS AF 6000 software. We took the pictures at the magnification 25X and 100X. The number of the cells in the song areas HVC, DLM, and DCN, which consist of lateral (DCNlat) and medial (DCNmed) parts, were calculated per mm^2^. All cells, including DAPI+, NeuN+, FoxP2+, PV+, DCX+, and BrdU+ were analyzed in these areas in a semi-automated way in the Fiji software. The function “Process—Find maxima” was applied to find the mean pixel intensity with the possibility of self-checking the number of counted cells due to an uneven focusing of the cells. Quantification of PNNs was obtained by the threshold adjust function in Fiji and characterized as a % of the given SCS area. We analyzed each hemisphere separately from at least 4 sections per SCS area for each type of the labeled cells. We took into account that the FoxP2 levels increase with the number of motifs sung before sacrifice [38], thus we normalized the number of FoxP2+ cells to the number of motifs sung before the perfusion.

Data were statistically analyzed using the Sigma plot 14.0 software. All comparisons were evaluated by two-way analysis of variance (ANOVA) followed by the Holm–Sidak post hoc test, multiple comparisons versus baseline group. The factors were the time from surgery and the brain region. For IHC, where we compared three time points, we used one-way ANOVA with the Holm–Sidak post hoc test, multiple comparisons versus baseline group. For DCX staining, where two time points were compared, the paired *t*-test was applied. The linear regression test or Pearson’s correlation test were used to find whether there are linear relationships between the pairs of variables.

## 3. Results

### 3.1. Area X Lesion Leads to a Temporary Decrease in the Number of Cells in HVC, DLM, and DCN

The spatial extent of the injury was assessed 8-10 days after Area X lesion based on the T2-weighted anatomical scans. We found that all the birds showed a hyper-intense signal caused by edema (Figure 1b) passing later on to a hypo-intense signal co-localized with Area X. The lesion size was 66.169 ± 3.972% (mean ± SEM) and it did not differ among the lesioned groups (*p* = 0.575; one-way ANOVA).

**Figure 2:**
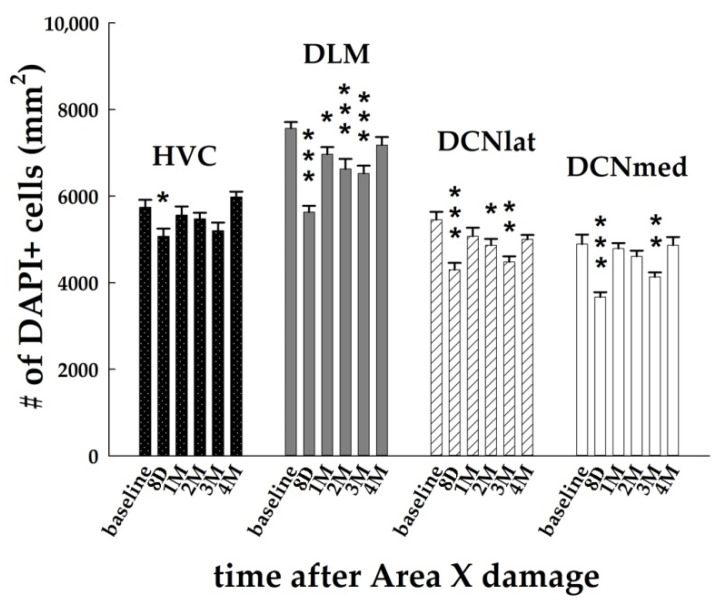
Quantification of the DAPI+ nuclei after Area X lesion in HVC, DLM, DCNlat, and DCNmed. Each bar represents mean and SEM. Two-way ANOVA was followed by the Holm–Sidak test with multiple comparisons vs. baseline group. * *p* < 0.05, ** *p* < 0.01, *** *p* < 0.001.

Our investigation of the changes occurring in the brain after the striatal injury started with the quantification of the cell nuclei in HVC DLM, DCNlat, and DCNmed (Figure 2). Two-way ANOVA with factors time (baseline, 8D, 1M, 2M, 3M, 4M) and brain region (HVC, DLM, DCNlat, DCNmed), followed by the Holm–Sidak test of multiple comparisons versus the baseline group revealed significant effects of both factors (*p* < 0.001 for time; *p* < 0.001 for brain region) on the number of DAPI+ cells.

**Figure 3:**
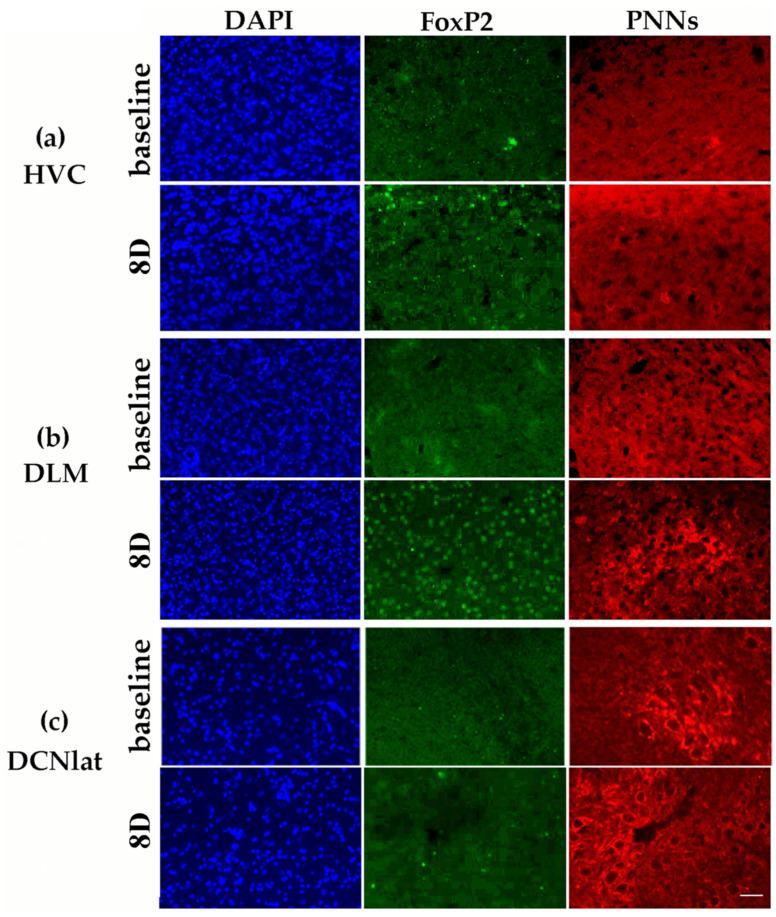
Immunohistochemical visualization of DAPI (blue), FOXP2 (green), and PNNs (red) in HVC (**a**), DLM (**b**), and DCNlat (**c**). The upper rows in a, b, and c illustrate the baseline group, and the bottom rows illustrate the group 8D after Area X damage. The images are taken from the center of the brain nuclei. The scale bar represents 50 µm.

Our hypothesis was that there will be changes downstream to damaged Area X, more probably than in the upstream areas connected with Area X. However, we found alterations in the number of DAPI+ nuclei also in HVC, where it decreased significantly at 8D post-lesion in comparison to the baseline group (*p* = 0.047; Figure 2 and Figure 3a). At 1M after the Area X lesion, the number of DAPI+ nuclei in HVC increased back to the baseline level (*p* = 0.442, Figure 2).

**Figure 4:**
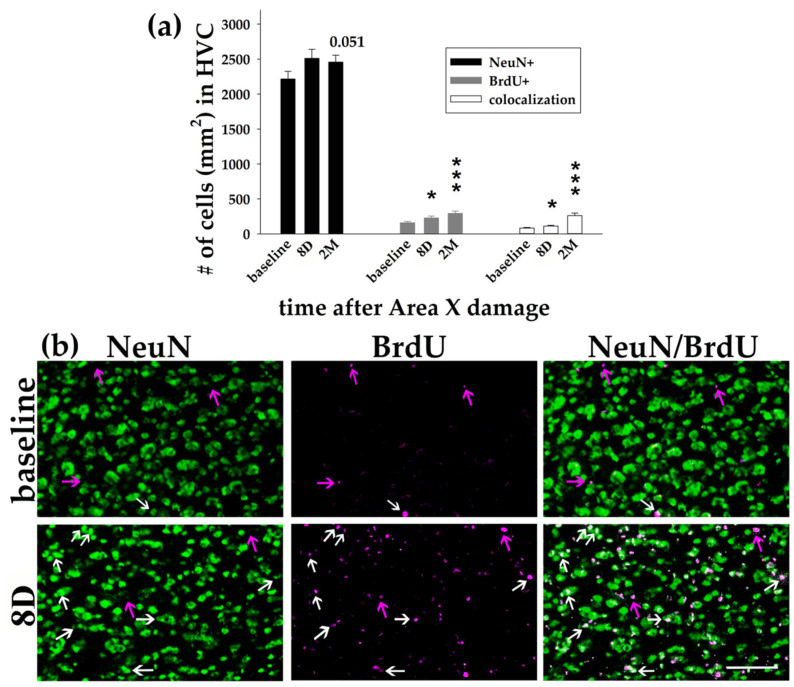
Neurons and newborn cells/neurons in the song nucleus HVC. (**a**) Quantification of the NeuN+ neurons, BrdU+ cells, and their co-localization in HVC of baseline birds, at 8D and 2M after Area X lesion. Each bar represents mean and SEM. *T*-tests for comparisons to the baseline. * *p* < 0.05, *** *p* < 0.001. (**b**) Examples of immunohistochemical staining showing NeuN (green) and BrdU (magenta) in a baseline bird (upper row) and a bird 8D after Area X lesion (bottom row). The white arrows point to the new BrdU+/NeuN+ neurons, the magenta arrows point to BrdU+ cells. The scale bar represents 50 µm.

The significant decrease in the number of DAPI+ cells in HVC at 8D (Figure 2) led us to specify whether it was due to the decline in the number of adult neurons (NeuN+) or it might be related to a decline in the incorporation of newborn cells (BrdU+). On the contrary, we found that the number of NeuN+ neurons in HVC was slightly elevated at 8D (*p* = 0.083, *t*-test) and 2M (*p* = 0.051, *t*-test) (Figure 4). We additionally found the increased number of BrdU+ cells at 8D (*p* = 0.046, *t*-test) as well as at 2M (*p* < 0.001; *t*-test) in comparison to the baseline (Figure 4). The number of co-localized BrdU+/NeuN+ neurons in HVC increased over time (*p* = 0.032 for 8D; *p* < 0.001 for 2M; *t*-tests; Figure 4).

Furthermore, we looked at the numbers of DAPI+ cells in the nucleus DLM that receives axons from Area X. It was characterized by long-term declining numbers of DAPI+ nuclei at 8D (*p* < 0.001), 1M (*p* = 0.037), 2M (*p* < 0.001), and 3M (*p* < 0.001; Figure 2). The numbers returned to the baseline level at 4M after the Area X lesion (*p* = 0.115). The lateral and medial parts of DCN exhibited remarkably similar numbers of DAPI+ nuclei in the baseline group (DCNlat: 5453.7 ± 183.5, DCNmed: 4892.4 ± 219.4, Figure 2 and Figure 3). The number of cells after the Area X lesion decreased significantly in DCNlat at 8D (*p* < 0.001), 2M (*p* = 0.015) and 3M (*p* < 0.01), and in DCNmed at 8D (*p* < 0.001) and 3M (*p* = 0.007, Figure 2 and Figure 3). There is evidence that DCN receives new neurons in mammals [56]. Therefore, we quantified the number of BrdU+ cells in both cerebellar nuclei. We found a significant increase of BrdU+ cells in DCNlat at 8D (*p* = 0.037) and at 3M (*p* = 0.045) after the Area X lesion. Similarly, the number of BrdU+ cells in DCNmed increased significantly at 8D (*p* = 0.009) and 3M (*p* = 0.022) after the Area X lesion. The intensity of NeuN staining was too inconsistent in DLM, DCNlat, and DCNmed and thus could not be quantified.

In summary, we observed an immediate decline in the number of DAPI+ nuclei in HVC, and a long-term decline in DLM and in both DCN for up to 3M after the Area X damage (Figure 2 and Figure 3). In all cases, the number of DAPI+ nuclei returned to the baseline level at 4M. It cannot be explained by the neuronal loss or by the decreased incorporation of the newborn cells because we found the opposite effect.

### 3.2. The Expression of FoxP2 after Area X Lesion Shows a Bi-Phasic Increase

Since FoxP2 expression is behaviorally regulated by undirected singing [29,38], we normalized the FoxP2 expression data to avoid the influence of singing. Two-way ANOVA with factors time and brain region revealed significant effects of both factors (*p* < 0.001 for time; *p* < 0.001 for brain region; *p* < 0.001 for their interaction) on the number of FoxP2+ cells.

**Figure 5:**
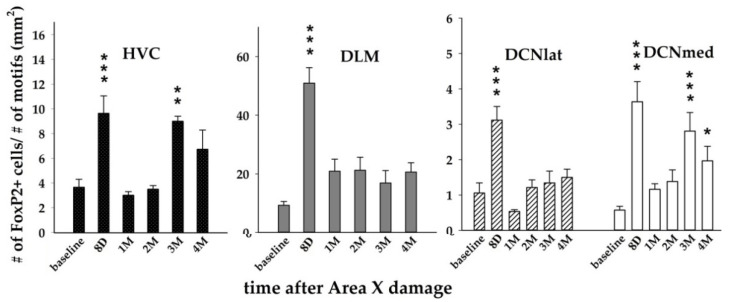
Quantification of the number of FoxP2+ cells in HVC, DLM, DCNlat, and DCNmed after Area X lesion. The number was normalized to the number of song motifs sung before the sacrifice. Each bar represents mean and SEM. Two-way ANOVA was followed by the Holm–Sidak test with multiple comparisons vs. the baseline group. * *p* < 0.05, ** *p* < 0.01, *** *p* < 0.001.

The following Holm–Sidak test comparing baseline with lesioned groups showed that the number of FoxP2+ cells increased significantly at 8D after the Area X lesion in HVC (*p* < 0.001), DLM (*p* < 0.001), DCNlat (*p* < 0.001), and DCNmed (*p* < 0.001, Figure 3 and Figure 5). The numbers of FoxP2+ cells were significantly higher also at 3M in HVC (*p* = 0.003) and DCNmed (*p* < 0.001), and at 4M in DCNmed (*p* = 0.021, Figure 5). Since FoxP2 was found to contribute to brain development, to promoting plasticity, and is expressed in young neurons in the neurogenic ventricular zone [27,33,45,57,58], we performed staining for the number of young (up to 30 days old) neurons expressing DCX [59] in baseline and 3M birds. We found the new DCX+ neurons in HVC but not in DLM, DCNmed, or DCNlat. Although the baseline group and 3M group in HVC did not differ in the number of DCX+ neurons (*p* = 0.510, *t*-test), the number of DCX+ neurons co-localizing with FoxP2 at 3M compared to the baseline group increased significantly (*p* = 0.042). There was also a positive linear regression relationship between the number of DCX+ and FoxP2+ cells (*p* = 0.022, R = 0.361, linear regression).

### 3.3. The Striatal Damage Affects PNNs Expression in Song Control System and Deep Cerebellar Nuclei

Since the song of zebra finches after Area X lesion becomes faster, more variable, and increases syllable repetitions [9], next we measured the expression of chondroitin sulfate proteoglycans that are part of the perineuronal nets (PNNs) suggested to play a role for song stabilization [50,54]. Thus, we would expect to find decreased levels of PNNs after Area X damage. Two-way ANOVA with factors time and brain region showed significant effects of both factors (*p* < 0.001 for time; *p* < 0.001 for brain region; *p* < 0.001 for the interaction) on the PNNs expression.

**Figure 6:**
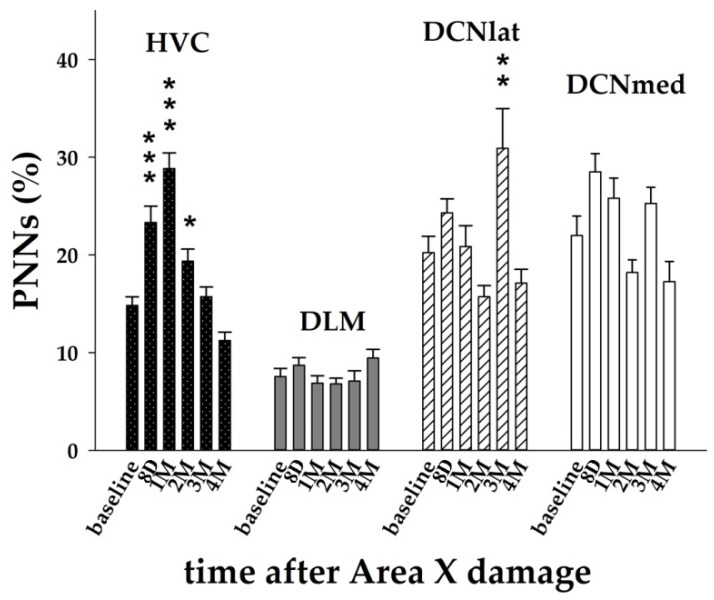
The expression of PNNs in HVC, DLM, DCNlat, and DCNmed after Area X lesion. Each bar represents mean and SEM. Two-way ANOVA was followed by the Holm–Sidak test with multiple comparisons vs. baseline group. * *p* < 0.05, ** *p* < 0.01, *** *p* < 0.001.

The Holm–Sidak post hoc test showed that the PNNs levels changed mostly in the song motor nucleus HVC. There was significantly increased expression of PNNs immediately after the lesion for up to 2M, with the peak at 1M (Figure 3 and Figure 6). We also found significantly increased PNNs expression in DCNlat at 3M (Figure 6). PNNs are associated with GABAergic inhibitory interneurons in HVC [60] that express PV [61,62]. Therefore, we examined whether the number of PV+ neurons changed together with the PNNs expression. As expected, we found that the number of PV+ neurons in HVC at 1M after the Area X lesion was significantly higher in comparison to the baseline (*p* = 0.015, *t*-test) and it returned to baseline levels at 4M (*p* = 0.287). Similar to HVC, also the increase of PNNs expression in DCNlat at the 3M post-lesion was accompanied by the increase of the number of PV+ neurons (*p* < 0.001; *t*-test). When we compared the number of PV+ cells and PNNs expression in the baseline, 8D, and 3M groups, we confirmed a positive linear relationship (*p* = 0.028, R = 0.225, linear regression).

## 4. Discussion

A neurotoxic lesion to the striatal vocal nucleus Area X in adult zebra finches leads to changes within the lesioned area, in the downstream target in the dorsal thalamus, and also beyond the traditional parts of the song system, in the cerebellum [20]. Although the technique of diffusion tensor imaging (DTI) was able to identify the location and time-course of the alterations, it was not able to determine their exact nature. Here, we identified some of the underlying cellular and gene expression changes.

While the immediate changes in the lesioned area are related to the excitotoxic damage and/or the inflammation, the later changes can be related to the recovery of Area X [9,23]. MRI in this study demonstrated that the lesion covered about 66% of Area X at 8-10 days post-surgery. Our T2-weighted images showed hyper-intense signals with features of edema within the injured area during the first days after injury. This was due to the high water content inside the lesion and a prolonged T2 relaxation time in comparison to healthy tissue. The same effect was described in other studies on much larger animals [63,64,65]. Inflammation following an insult is necessary to assist in clearing damage and preparing for neuronal circuits remodeling [66]. These changes include massive responses of astrocytes and microglia [67]. As reported in the previous study with zebra finches [20], there were no visible traces of edema in Area X 2 weeks after the excitotoxic injury. In accordance, the edema already disappeared in the 1M scans in this study, and the lesion size was reduced. This corresponds to the dynamic process of Area X recovery, where the most pronounced regeneration was observed during the first month after the injury [9,23]. The micro and macrostructural changes found by DTI occurred in DTZ in the location of the vocal nucleus DLM directly connected to Area X as well as in the DCN of the cerebellum. Moreover, the lesion-induced song alterations correlated with some DTI parameters measured in and around HVC [20]. Therefore, we included all these three regions in our analyses.

The loss of synaptic inputs as well as synaptic targets after brain injury may cause distant neurons to undergo apoptosis [68]. Both DLM and HVC showed decreased numbers of cell nuclei (DAPI+) immediately after the injury. While in HVC it could also be caused by the inflammation of the injured Area X, it is probably not the case for DLM where the lower numbers of cells last for up to 3M. The decreased numbers of cell nuclei in DLM found in this study corresponds with the prolonged changes in DTZ found in the previous study by DTI [20]. Since the number of adult neurons (NeuN+) as well as newly arrived cells and neurons (BrdU+, BrdU+/NeuN+, DCX+) in HVC slightly increased, we suppose that the decrease in DAPI was due to the loss of glial cells. On the other hand, PNNs expression in HVC increased immediately after the striatal injury and stayed higher for up to 2M, with the peak at 1M. While astrocytes and microglia protect the intact tissue surrounding the injured region [69,70], PNNs also have a protective role from oxidative stress or stress in more distant regions [71]. We suppose that the role of PNNs in HVC during the first two months post-lesion is in stabilizing synaptic connectivity [72] more than in the formation of new synaptic connections [45,48,73]. PNNs expression was shown to be associated with the inhibitory interneurons in HVC expressing PV [61,62]. In songbirds, the increases in PNNs around PV+ neurons coincide with the closure of the sensitive period for song learning [49]. PV+ neurons have been shown to exhibit more stable synaptic transmission than other types of interneurons, and the plasticity of PV+ neurons in adults is supposed to be limited, for instance, by the presence of PNNs [74] that surround neuronal bodies and proximal neurites of PV+ neurons [47]. In accordance, we found that the PNNs increase in HVC in our study was accompanied by the increased number of PV+ cells.

Neurons within the striatal vocal nucleus Area X project to the thalamic nucleus DLM and both cerebellar nuclei, lateral and medial, send axons to the dorsal thalamus [10,13,75]. The cerebellum also modulates the activity in the striatum via the disynaptic pathway through the thalamus in mammals [76] and plays a major role in motor coordination and learning [77]. Neurons in DCN are surrounded by PNNs [78,79], which are diminished during associative motor learning in mice and restored when memories are fully acquired [78]. In our study, we found relatively high levels of PNNs expression in DCN, and it increased in lateral DCN at 3M after Area X damage. We suppose that the increased level of PNNs expression might promote synaptic stabilization after lesion intervention, which is necessary for the subsequent maintenance of motor traces memory. PNNs were previously associated with the closing critical period of learning [46,47], post-injury restoration [80], learning processes [81,82], and the consolidation of previously acquired motor memories [78]. These plasticity changes allow synapses to meet the specific functional and adaptive requirements of changing conditions.

Summarizing the data of PNNs in our study, PNNs seem to play a protective and consolidative role in HVC immediately after the Area X damage. The increased expression for up to 3M post-lesion suggests protection of neurons, which are incorporated in existing neural circuits. This might be crucial for the neurons that are not able to renew, such as the neurons projecting from HVC to Area X and the interneurons [61,83]. On the other hand, the later increase at 3M in the cerebellum can provide an accommodating environment for synaptic reconnection early after Area X damage.

FoxP2 on the neuronal level increases dendritic spine density [31] as well as synaptic plasticity and dendritic length [84,85]. Overexpression of FoxP2 in Area X dysregulated dopaminergic innervation and affects the song variability and the song maintenance [86,87]. Overexpression of FoxP2 accompanied by deafening accelerates song deterioration [88,89]. High FoxP2 expression in striatal medium spiny neurons may increase their capacity for receiving inputs [34]. In our study, FoxP2 expression showed an increase at 8D, following the injury in all measured areas, and another increase at 3M in HVC and DCNmed. Thus, the immediate increase can be possibly related to synaptic changes and the reorganization in the nuclei downstream and upstream to the damaged Area X due to the neurotoxic injury as well as the inflammation in Area X. Area X shows a massive regeneration at 2M after the neurotoxic lesion in more than 80% of the nucleus (this study [9,23]). We suppose that the later increase of FoxP2 in HVC and the cerebellum at 3M might be associated with this regeneration and the synapse formation.

Area X lesions lead to an immediate increase in song syllable repetition and changes in song tempo in adult zebra finches and Bengalese finches [9,21] and decreased within syllable variation in fundamental frequency [22]. The increased song syllable variability and syllable repetitions can be caused by the abolishment of FoxP2 after the neurotoxic injury in the striatum as the knockdown of FoxP2 in Area X of adult zebra finches leads to increased song syllable variability and syllable repetitions [90]. Intracellular recordings from LMAN neurons showed that FoxP2 knockdown in Area X accelerates signal propagation from HVC to LMAN and abolishes context-dependent modulation of LMAN activity as well as context-dependent changes in song variability [89].

Although the overall organization of mammalian and avian brains seems to be quite diverse in a structural way (laminal vs. nuclear organization) [91], there are similarities in neuronal circuitry and connections [15,92]. An important node to the striatal network in most mammals is the pulvinar nucleus of the thalamus. Previous studies have shown that pulvinar is mutually and extensively connected with the sensory cortex, superior colliculus, amygdala, and also striatum [93] and plays important roles in multi-sensory processing and defensive responses [94]. The dysfunction of the pulvinar-striatum circuit is associated with Parkinson’s disease [95]. The mammalian pulvinar nucleus resembles the avian *nucleus rotundus* in its input and projections [96]. Although this nucleus may be an interesting potential target for the changes following the striatal lesion, it is not known to be connected with the striatal vocal nucleus Area X in birds, and we did not observe any changes in this region in our previous [20] or presented study.

## 5. Conclusions

We tested whether the striatal excitotoxic lesion leads to significant changes in the expression of plasticity markers. All the areas identified by MRI in the previous study [20] showed modulation of neural plasticity after Area X damage and regeneration. Here, this plasticity was represented by the expression of FoxP2, PNNs, DCX, and other immunohistochemical markers, such as BrdU and DAPI. We showed that PNNs and FoxP2 exhibited dynamic expression patterns until 4M after the striatal lesion. The damage in the striatal vocal area led not only to the changes in the downstream thalamic target, but also caused substantial alterations in the upstream cortical area. Remarkably, the striatal impairment also modified gene expression in the cerebellum as well. Together, these results suggest some immediate and some long-term changes in the plasticity and protection of areas directly connected to the damaged striatum but also in areas distantly connected, such as the cerebellum.

## Data Availability

All the data used to support the findings of this study are included in the article. If further information is needed, it can be available from the corresponding author upon request.
